# Retrograde mesenteric perfusion from the deep femoral artery in a patient with a recurrent anastomotic aneurysm in the groin: a case report

**DOI:** 10.3389/fsurg.2023.1208871

**Published:** 2023-06-23

**Authors:** Benoit Geng, Markus Menth, Lucien Widmer, Bernhard Egger, Emmanouil Psathas

**Affiliations:** ^1^ Department of Surgery, Unit of Vascular Surgery, HFR Fribourg Cantonal Hospital, Fribourg, Switzerland; ^2^ Department of Radiology, HFR Fribourg Cantonal Hospital, Fribourg, Switzerland

**Keywords:** mesenteric ischemia, splanchnic collateral pathways, anastomotic aneurysm, deep femoral artery, retrograde mesenteric perfusion

## Abstract

In patients with atherosclerotic disease in two of the three main vessels in the splanchnic circulation, symptoms of chronic mesenteric ischemia may arise, depending on the disease chronicity and the presence of mesenteric collateral pathways. The most commonly described collateral pathways are between the celiac artery (CA), superior mesenteric artery (SMA), and inferior mesenteric artery (IMA); and between the IMA and the internal iliac artery (IIA). Another collateral network between the deep femoral artery and the IIA can also become important, especially in patients with aorto-iliac occlusion. Here we report a patient with a symptomatic anastomotic aneurysm of the right femoral artery after a previous aorto-bi-femoral bypass. This patient’s bowel viability relied on a well-developed collateral network from the ipsilateral deep femoral artery. This unusual anatomy required special surgical considerations and planning, to minimize the risk of perioperative mesenteric ischemia. During open repair, distal femoral debranching with a distal-to-proximal anastomotic sequence allowed minimizing of the ischemic time, and avoidance of potential ischemic complications from the visceral circulation. This case emphasizes the importance and benefit of the deep femoral artery and its collaterals as a reserve network of the splanchnic circulation. Favorable outcomes can be achieved with careful analysis of the preoperative imaging and proper planning, with adaptation of the surgical strategy.

## 1. Introduction

Within the splanchnic circulation, a rich and complex collateral arterial network protects the bowel from ischemia and infarction. This is especially important for individuals with atherosclerotic chronic occlusion of one or more of the main splanchnic arteries.

Here we report an unusual case in which a patient had an enlarging recurrent anastomotic aneurysm of the right femoral bifurcation, and concomitant complete chronic occlusion of all main splanchnic arteries. This patient’s bowel viability relied on a well-developed collateral network from the ipsilateral deep femoral artery.

## 2. Case description

A 74-year-old female patient was referred to our department due to an enlarging tender pulsatile mass of the right groin. Comorbidities included hypertension, hyperlipidemia, chronic obstructive pulmonary disease (GOLD stage III), and current smoking. The patient’s medical history included radical esophagectomy and esophageal reconstruction with a gastric tube for carcinoma of the thoracic esophagus, 27 years earlier; transient ischemic attack, 22 years earlier; and *in situ* squamous cell carcinoma of the glottic larynx, 5 years earlier, for which she had undergone radio-chemotherapy with subsequent complete remission. Additionally, 21 years earlier, the patient had undergone aorto-bi-femoral (ABF) bypass for severe bilateral claudication due to extensive aortoiliac occlusive disease. Nine years after that bypass, she had presented with bilateral groin anastomotic aneurysms that were treated by replacement of both ilio-femoral parts of the bifurcated aortic graft.

Clinical examination revealed a 40-kg sarcopenic patient, with a large pulsatile mass of the right groin. The mass was tender upon palpation, with no signs of cutaneous inflammation, suggesting a symptomatic aneurysm.

## 3. Diagnosis and treatment

**Figure 1:**
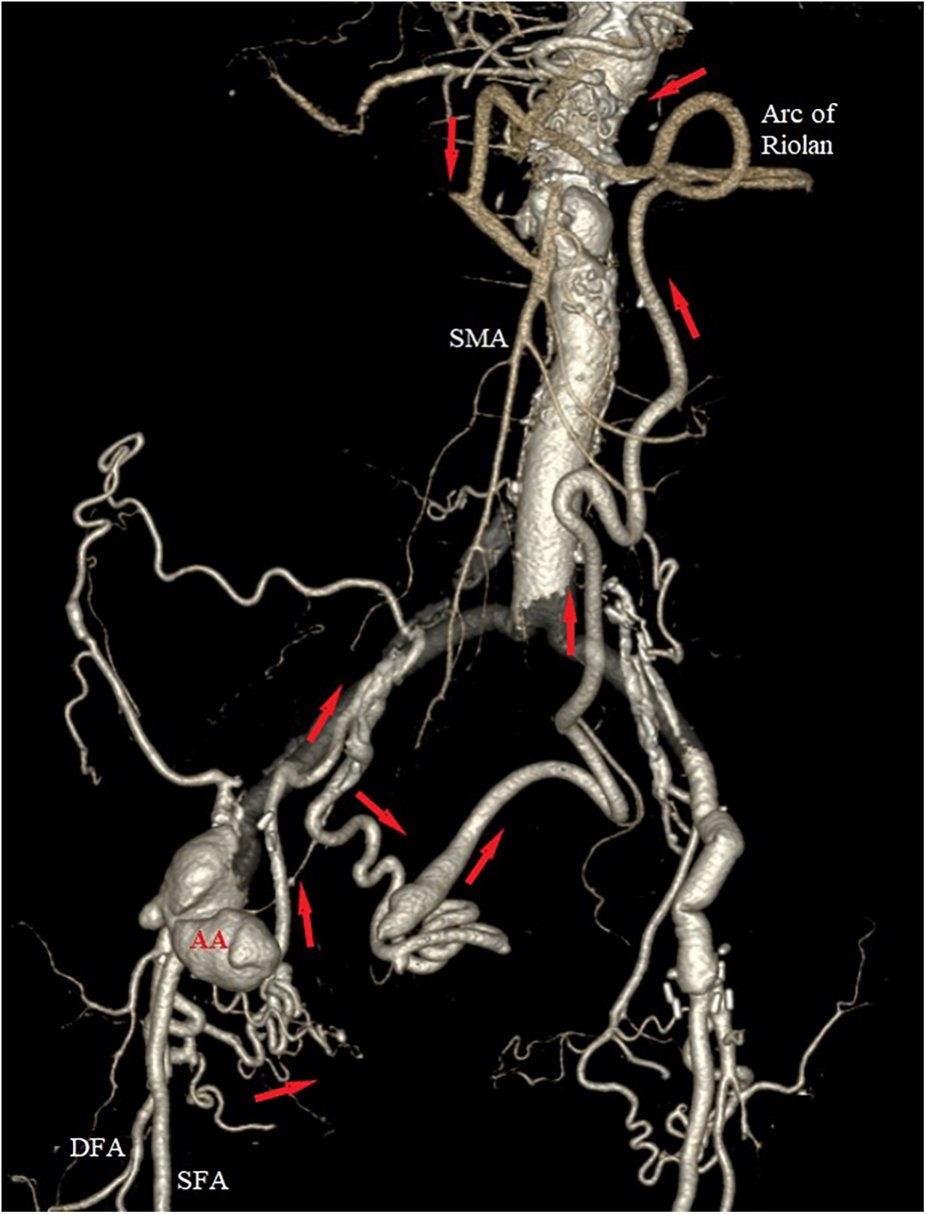
Preoperative 3D volume rendering of the computed tomography angiography (CTA), showing the anastomotic aneurysm (AA) of the right groin, with retrograde perfusion of the superior mesenteric artery (SMA) via a highly developed collateral network (red arrows) from the right deep femoral artery (DFA). Notice the ostial occlusion of the right hypogastric, the inferior and superior mesenteric arteries, and the well-developed arc of Riolan.

Computed tomography angiography (CTA) was immediately performed, which confirmed the presence of a 5.6-cm anastomotic aneurysm of the right common femoral artery (CFA), with infiltration of the surrounding soft tissue. No extravasation of contrast agent was apparent, indicating that rupture was not occurring. Further careful examination of the CTA revealed complete occlusion of the celiac artery (CA), superior mesenteric artery (SMA), inferior mesenteric artery (IMA), and right renal artery; focal aortic dissection above the proximal graft anastomosis; bilateral ostial occlusion of both internal iliac arteries (IIAs); and mild stenosis of the left renal artery. The right deep femoral artery (DFA) originated from the aneurysmal sac, and presented with a complex network of collaterals proximally passing through the obturator canal to the anterior distal part of the right IIA. Following the posterior branch of the right IIA, a well-developed collateral network was apparent via the superior and middle rectal arteries, providing retrograde perfusion of the IMA, which itself provided flow to the SMA via the arc of Riolan and the left branch of the middle colic artery (Figure 1). Interestingly, the patient reported no signs of abdominal angina, e.g., postprandial abdominal pain or weight loss. She was accustomed to eating frequent small meals to avoid reflux and diarrhea after her radical esophagectomy.

**Figure 2:**
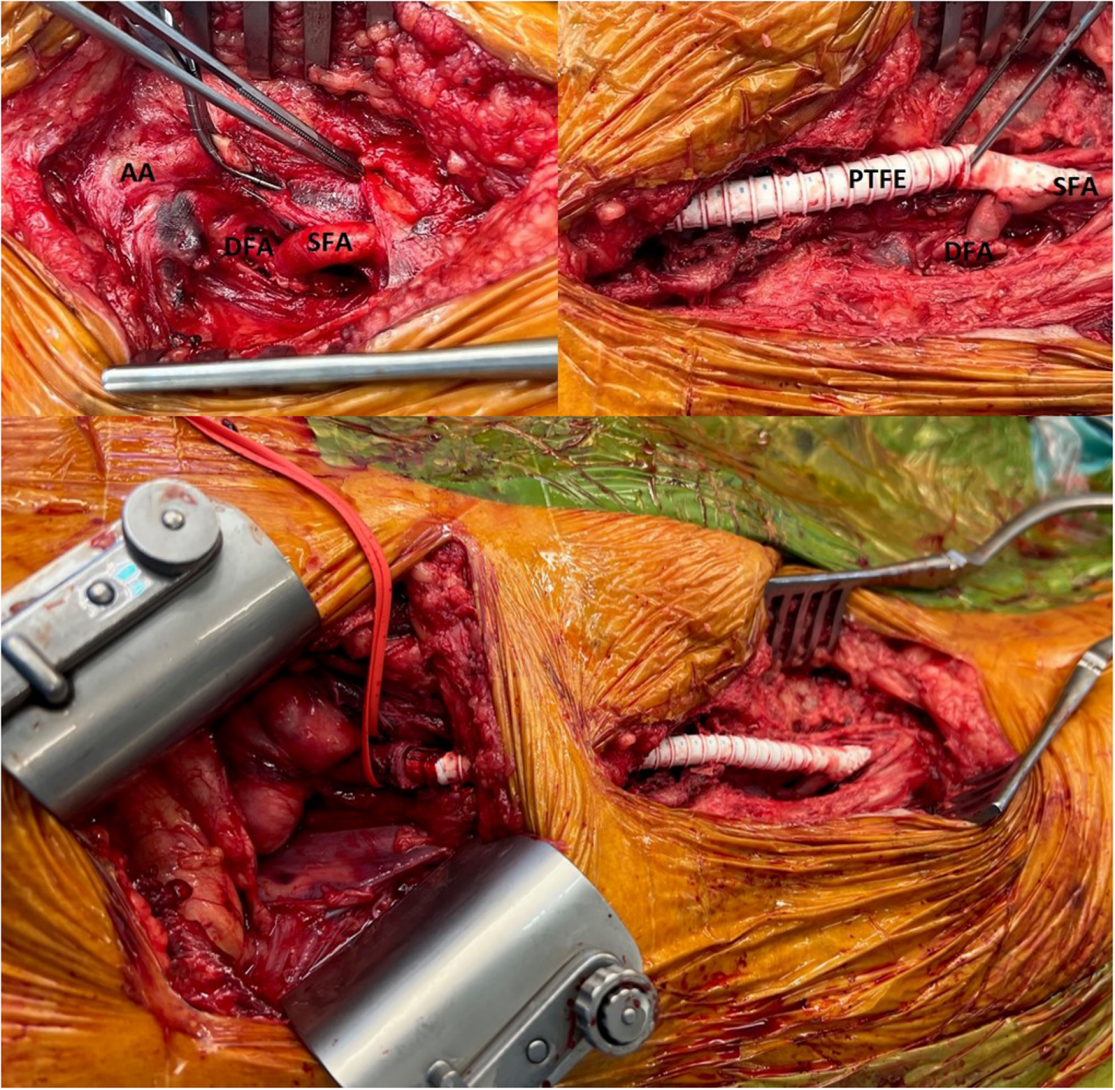
Intraoperative view of the right groin. Top Left: After distal debranching and re-implantation of the proximal superficial femoral artery (SFA) to the distal deep femoral artery (DFA). AA, anastomotic aneurysm. Top Right: After distal end-to-side anastomosis of a PTFE ilio-femoral graft to the de-branched superficial femoral artery. Bottom: Operative view of the final reconstruction.

Due to the symptomatic nature, and the radiological findings of anastomotic aneurysm, emergent surgical repair was initiated. Under general anesthesia, the proximal right limb of the ABF graft was controlled via a right retroperitoneal approach; while the femoral aneurysm, proximal superficial femoral artery (SFA), and distal DFA were dissected through a longitudinal groin incision. To minimize the risk of perioperative mesenteric ischemia due to clamping of the proximal DFA, the DFA and the proximal part of the SFA were dissected approximately 5 cm after their origins and controlled using vessel loops. After systematic administration of 100 IU/kg of heparin, the proximal SFA was transected and re-implanted on the distal DFA, while maintaining perfusion of its proximal part. Next, a distal end-to-side anastomosis was performed between the re-implanted SFA and an 8-mm PTFE graft (Figure 2). After completion of both anastomoses, the right limb of the ABF graft was clamped, the aneurysmal sac was opened, and the ostium of the DFA within the sac was closed using a 4-0 monofilament suture. Then the proximal end of the PTFE interposition graft was retrograde tunneled to the right retroperitoneal region, and a proximal end-to-end anastomosis performed. After a total ischemia time of 18 min, the right limb was re-perfused by opening the clamps. All arterial branches were controlled for patency and adequate Doppler signals, followed by closure of the access incisions.

Postoperatively, the patient was transferred to the intensive care unit. While there, she unfortunately developed a retroperitoneal hematoma, requiring surgical revision and drainage. The postoperative course was otherwise uneventful. She was discharged home on postoperative day 10. The patient was prescribed long-term antithrombotic therapy with low-dose aspirin (100 mg/day) combined with rivaroxaban (2.5 mg twice daily).

**Figure 3:**
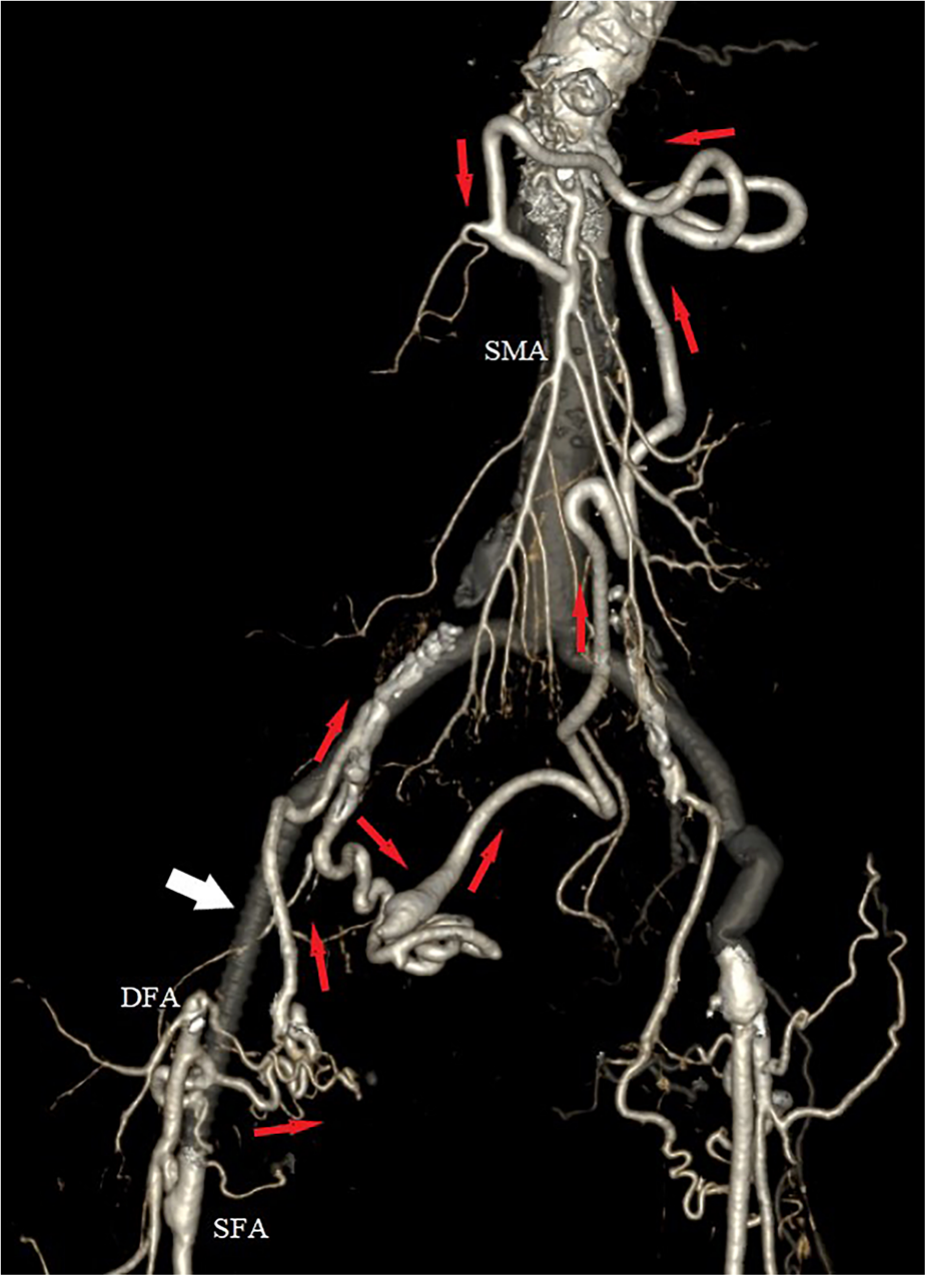
Postoperative 3D volume rendered computed tomography angiography (CTA), with exclusion of the aneurysm and preservation of the collateral network (red arrows) between the right deep femoral artery (DFA) and the superior mesenteric artery (SMA). White arrow indicates the interposition of the PTFE iliofemoral graft.

At her 6-month outpatient follow-up, the patient remained asymptomatic. A control CTA revealed a patent vascular reconstruction, with preservation of the collateral network between the DFA and the splanchnic vessels. However, the collaterals passing through the deep circumflex iliac artery had to be sacrificed (Figure 3).

## 4. Discussion

In patients with atherosclerotic disease in two of the three major vessels of the splanchnic circulation, symptoms of chronic mesenteric ischemia may arise, depending on the disease chronicity and the presence of mesenteric collateral pathways. The most important collateral pathway connecting the celiac artery with the superior mesenteric artery passes through the pancreaticoduodenal arterial arcade. On the other hand, the arc of Riolan and the marginal artery of Drummond connect the SMA to the IMA via anastomoses of the left branch of the middle colic artery with the ascending branch of the right colic artery in the splenic flexure. Finally, the IMA and the ILA are connected via collateral arcades involving the superior and middle rectal arteries. Another more distal collateral network exists between the deep femoral artery and the internal iliac artery, which is particularly important in cases of chronic aorto-iliac occlusions (1, 2).

The unusual arterial anatomy presented in this case report serves as an excellent reminder of the importance of these collateral networks in patients with advanced atherosclerotic disease and prior aortic surgery. It is obvious that the IMA was not re-implanted during the index aortic operation 27 years previously, while the SMA and the CA became gradually occluded over the years. However, our patient never developed symptoms of acute or chronic mesenteric ischemia. Collaterals from the contralateral DFA and IIA participated in the retrograde perfusion of the SMA, but radiological findings indicated that they were more developed on the ipsilateral side, which also presented a symptomatic anastomotic aneurysm.

We discussed the option of prophylactic visceral revascularization prior to surgical repair of the groin aneurysm; however, this approach was not chosen because the patient was asymptomatic despite the three-vessel occlusion. Due to the special importance of the DFA and its origin from the aneurysm sac, a total endovascular or hybrid approach would probably compromise its patency, without significantly reducing perioperative morbidity. Instead, we decided to take all necessary measures to preserve the collaterals, and to reduce clamping time of the proximal DFA as much as possible. This was achieved by performing initial distal femoral debranching, and distal-to-proximal anastomoses of the iliofemoral interposition graft. It may also have been possible to use a temporary femoro-femoral or ilio-femoral shunt, as described by Osterberg et al. (3); however, the required material was not readily available in our hospital at that moment. Furthermore, there was probably not enough space for retrograde placement of a sheath in the distal DFA.

The deep femoral artery is known to be important for maintaining adequate limb perfusion in patients with femoro-popliteal occlusions and critical limb ischemia. However, the role of the DFA as a second- or third-line backup collateral network in the visceral circulation has not yet been adequately described. The presently described unusual case provides an excellent illustration of the added clinical value of this deep femoral artery collateral network. Favorable outcomes can be achieved through careful analysis of the preoperative imaging and proper planning, with adaptation of the surgical strategy.

## Data Availability

The original contributions presented in the study are included in the article, further inquiries can be directed to the corresponding author.
